# Left ventricular diastolic dysfunction as a mediator between the CHA_2_DS_2_-VASc score and left atrial strain

**DOI:** 10.1186/s44348-026-00097-2

**Published:** 2026-09-17

**Authors:** Yu Jin Chung, Ji-won Hwang

**Affiliations:** Division of Cardiology, Department of Medicine, Konkuk University Medical Center, Seoul, Republic of Korea

## Abstract

The CHA₂DS₂-VASc score, originally developed for stroke risk stratification in atrial fibrillation, comprises cardiovascular risk factors that promote left ventricular (LV) diastolic dysfunction and left atrial (LA) remodeling. In this issue, Mahmoud et al. examined the association between the CHA₂DS₂-VASc score and LA function assessed by speckle-tracking echocardiography in 150 patients in sinus rhythm. LA reservoir and conduit strain decreased progressively with increasing risk category, whereas LA volume index did not differ significantly, indicating that functional impairment preceded overt LA enlargement. Using mediation analysis, the authors showed that the association with conduit strain was largely mediated by LV diastolic dysfunction, whereas reservoir strain retained a smaller direct association, suggesting an intrinsic atrial component consistent with atrial cardiomyopathy. This commentary discusses these findings within the framework of atrial remodeling and contemporary echocardiographic assessment, considers the study's principal limitations, and highlights the need for longitudinal studies to determine the prognostic significance of subclinical LA dysfunction in patients without atrial fibrillation.

The CHA_2_DS_2_-VASc score was developed to refine stroke and thromboembolic risk stratification in patients with atrial fibrillation (AF), and it remains the standard tool recommended for this purpose [1]. Beyond its original application, the score has also been associated with adverse events in populations without AF, including patients with heart failure who remain in sinus rhythm, reflecting the composite of established cardiovascular risk factors [2]. The individual components of the score—hypertension, diabetes, older age, and vascular disease—are each known to promote left ventricular (LV) diastolic dysfunction and left atrial (LA) remodeling, thereby raising the question of whether the score may reflect subclinical LA dysfunction in patients who have not yet developed AF.

The left atrium contributes to LV filling through three sequential functions: reservoir during ventricular systole, conduit during early diastole, and booster pump during late diastole [3]. Speckle-tracking echocardiography (STE) allows quantification of LA myocardial deformation throughout these phases and can detect functional impairment before LA enlargement becomes evident on conventional volumetric measurement [4]. Reduced LA strain has been reported in patients with higher CHA_2_DS_2_-VASc scores, including those in sinus rhythm awaiting coronary artery bypass surgery [5]. However, whether the score is associated with LA dysfunction independently of LV diastolic dysfunction or entirely through it remains unclear.

In this issue of *Journal of Cardiovascular Imaging*, Mahmoud et al. [6] investigated the association between the CHA_2_DS_2_-VASc score and LA function assessed by STE in 150 patients in sinus rhythm stratified into low-, moderate-, and high-risk groups. LA reservoir strain (LASr) and LA conduit strain (LAScd) decreased progressively with increasing risk category, whereas the maximal LA volume index did not differ significantly among the groups. This pattern suggests that functional impairment measured by strain preceded overt LA enlargement. After adjustment for age, differences in mitral inflow parameters (E wave, A wave, and E/A ratio) were no longer significant, whereas isovolumic relaxation time, deceleration time, e′, and E/e′ remained different among the groups, indicating that some of the observed differences reflected physiological aging rather than the CHA_2_DS_2_-VASc score itself.

A distinct feature of the study is the use of mediation analysis to examine the pathway underlying the observed association. For LAScd, its association with the score was largely mediated by LV diastolic dysfunction (approximately 90%), with no significant residual direct effect. This finding is consistent with the passive nature of the conduit phase, which is sensitive to LV filling pressure. For LASr, a smaller but significant direct association persisted after accounting for diastolic function, which the authors interpret as a possible marker of intrinsic atrial pathology, such as fibrosis or reduced compliance, consistent with the concept of atrial cardiomyopathy [7]. This distinction is clinically relevant because it differentiates impairment secondary to LV diastolic dysfunction from impairment that may originate within the atrium itself.

Several limitations should be considered. The cross-sectional design does not permit causal or prognostic inferences, and longitudinal studies are needed to determine whether reduced LA strain predicts incident AF, stroke, or heart failure in this population. The single-center cohort was relatively small for a mediation model. LA strain was analyzed using LV-dedicated software rather than a dedicated atrial module, and intervendor and intersoftware differences in absolute strain values are recognized; however, the authors mitigated this limitation by consistently using a single platform [8]. In addition, E/e′ was used as the sole surrogate of diastolic function [9], and the estimated mediated proportions may vary depending on the selected mediator. Of note, the 2025 update on the evaluation of LV diastolic function from the American Society of Echocardiography has since incorporated LA strain into the assessment algorithm, which may further refine the relationship examined here [10]. Finally, the study was designed using the CHA_2_DS_2_-VASc score, whereas the 2024 European Society of Cardiology guidelines have since adopted the sex-neutral CHA_2_DS_2_-VA score, which should be considered when applying these group definitions to current clinical practice[11].

In summary, Mahmoud et al. [6] demonstrate that a higher CHA_2_DS_2_-VASc score is associated with lower LASr and LAScd in patients in sinus rhythm and that this association is largely, though not entirely, mediated by LV diastolic dysfunction. These findings support the value of STE in detecting early LA functional impairment and suggest that the CHA_2_DS_2_-VASc score may reflect atrial and ventricular remodeling before the development of AF. Further studies incorporating longitudinal follow-up, dedicated atrial strain software, and complementary tissue characterization by cardiac magnetic resonance would help clarify the clinical significance of these observations for risk stratification.

## Data Availability

No datasets were generated or analysed during the current study.

## Abbreviations

AF: Atrial fibrillation
EACTS: European Association for Cardio-Thoracic Surgery
LA: Left atrial
LAScd: Left atrial conduit strain
LASr: Left atrial reservoir strain
LV: Left ventricular
STE: Speckle-tracking echocardiography
